# Exploring three-dimensional right ventricular shape and function in Tetralogy of Fallot patients

**DOI:** 10.1007/s10554-026-03781-y

**Published:** 2026-07-29

**Authors:** Robert R. Zwaan, Yue Chen, Jop W. Schneijdenberg, Daniel J. Bowen, Robert M. Kauling, Gerard van Burken, Johan G. Bosch, Annemien E. van den Bosch

**Affiliations:** 1 Department of Cardiology, Cardiovascular Institute, Thorax Center, Erasmus MC, Rotterdam, the Netherlands; 2 European Reference Network for Rare, Low Prevalence and Complex Diseases of the Heart (ERN GUARD-Heart), Amsterdam, The Netherlands

**Keywords:** Tetralogy of Fallot, Right Ventricle, Three-Dimensional Echocardiography, RV remodeling

## Abstract

Tetralogy of Fallot (ToF) accounts for approximately 7–10% of all congenital heart disease and is frequently complicated by right ventricular (RV) dysfunction. Three-dimensional echocardiography (3DE) offers more accurate assessment of RV volume and function, facilitating advanced evaluation of RV geometry. Building on this, a custom software tool was developed to quantify RV shape and function. This study aimed to assess the differences of 3DE derived RV shape and function parameters in ToF and healthy controls. 3DE RV datasets were acquired in 50 ToF and 50 healthy controls. RVs were segmented using commercially available software (TomTec) and further processed with custom software to quantify mean curvature (MC), area strain (AS), and longitudinal strain (LS). Measurements were performed globally and regionally across predefined RV segments. Curvature analysis identified the posterior boundary (253% [227–265] vs. 283% [260–301] vs. 294% [264–329]; for volume overload, absent overload and healthy controls, respectively; *p* < 0.001) and apex (464% [419–529] vs. 501% [467–541] vs. 554 [486–619]; for volume overload, absent overload and healthy controls, respectively; *p* < 0.001) as key regions driving RV remodeling in ToF. Septal flattening was observed in ToF with decreased concavity of the SB (*p* = 0.007). Global RV AS was significantly reduced in ToF (*p* < 0.05), with the largest impairment observed at the apex (-16% [-21;-12] vs. -14% [-17;-6] vs. -38% [-42;-28]; for volume overload, absent overload and healthy controls, respectively; *p* < 0.001). This in-house developed software enables comprehensive RV morphology and deformation analysis from standard 3DE imaging. Curvature analysis provides a novel and sensitive measure of RV remodeling, while AS and LS offer detailed insight into regional RV dysfunction. Improved characterization of RV remodeling may support clinical decision-making.

**Graphical abstract:**

**Figure:**
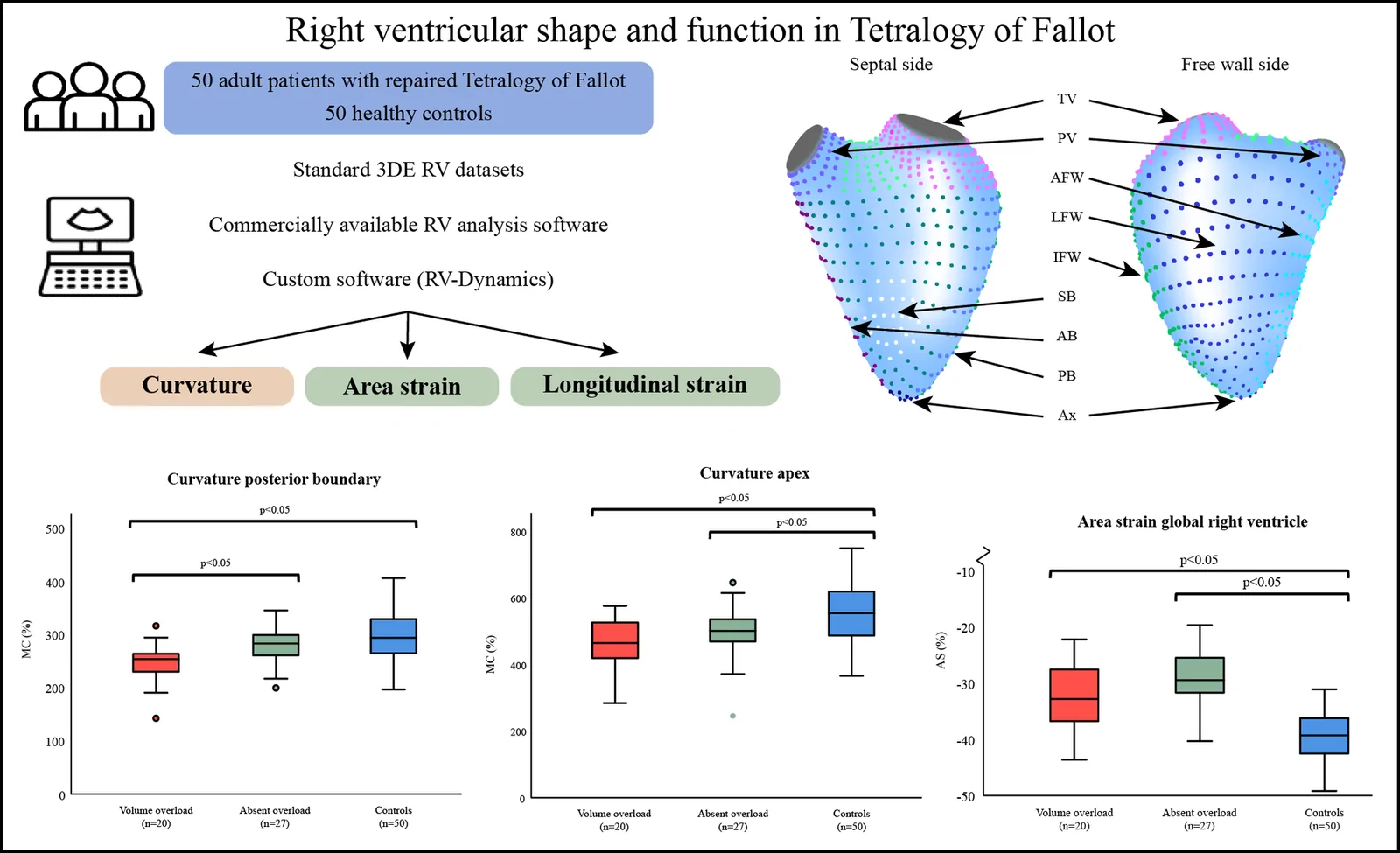

**Supplementary Information:**

The online version contains supplementary material available at https://doi.org/10.1007/s10554-026-03781-y.

## Introduction

Tetralogy of Fallot (ToF) is the most common cyanotic congenital heart disease (CHD) and accounts for approximately 7–10% of all CHD cases [1, 2]. Advances in surgical techniques have resulted in excellent long-term survival, with up to 95% of patients reaching adulthood. Despite improved survival, late morbidity remains substantial. Compared with patients with simpler congenital defects, ToF patients face a higher risk of arrhythmias, heart failure, and sudden cardiac death, highlighting the need for lifelong surveillance [3–6]. 

Pulmonary regurgitation (PR) following transannular patch repair is considered a major contributor of late complications in ToF. Chronic PR induces right ventricular (RV) volume overload, leading to adverse RV remodeling as a compensatory mechanism to normalize wall stress. While initially well tolerated, prolonged volume overload may result in irreversible RV dysfunction, arrhythmias, and increased mortality [7–9]. 

Pulmonary valve replacement (PVR) plays a central role in preventing irreversible RV damage. However, determining the optimal timing of PVR remains clinically challenging. Accurate imaging of RV size, shape, and function is essential for guiding management, yet is complicated by the RV’s complex geometry and anterior position. Conventional two-dimensional echocardiographic parameters rely on geometric assumptions and single-plane assessments, limiting their ability to capture the three-dimensional (3D) nature of RV remodeling.

Three-dimensional echocardiography (3DE) offers more accurate and comprehensive assessment of RV volume and function, hereby paving the way for advanced RV evaluation. Building on this technology, we developed a custom software tool that utilizes standard 3DE RV datasets to quantify regional RV shape and function. In this study, we aim to evaluate differences in 3DE derived RV morphology and deformation between adults with repaired ToF and healthy controls. Improved characterization of RV remodeling may enhance understanding of RV failure mechanisms and improve clinical decision-making.

## Methods

### Study population and design

Adult patients with repaired ToF were identified from research cohorts at our center (MEC-2010-165, MEC-2018-1541, MEC-2019-0465 and MEC-2025-0153). Patients who had undergone 3D transthoracic echocardiography (TTE) were reviewed by an experienced echocardiographer (DB). Individuals with moderate-to-good 3D RV image quality, defined as those with analysable RV mesh reconstructions (‘*Beutels*’), were considered eligible for inclusion. An age-matched control group consisted of healthy volunteers who had previously undergone 3D RV echocardiography at our center (MEC-2013-447, MEC-2016-427). More detailed description is available in previously published work [10, 11]. The study was conducted in accordance with the principles of the Declaration of Helsinki and was approved by the local medical ethical committee.

### Three-dimensional echocardiography

All 3DE recordings were acquired from the apical window during a single breath hold using an EPIQ7 ultrasound system (Philips Medical Systems, Best, the Netherlands) equipped with an X5-1 matrix array transducer. Acquisitions consisted of either four to six beat full volume datasets, or single beat datasets obtained using HeartModel software (Philips Healthcare). Recordings were optimised to include the RV at the highest possible volume rate, with slight over-gaining of the two-dimensional (2D) image. The 3D datasets were digitally exported and analysed using specialised commercially available RV analysis software (4D RV-Function 2.0, TomTec Imaging GmbH, Unterschleissheim, Germany). Set anatomical landmarks were placed, after which all contours were checked and manually adjusted when necessary.

### RV-dynamics

3D dynamic RV surface renderings were exported from TomTec as a set of triangulated mesh files, one per frame of the dynamic 3DE study, and used as input for the in-house developed RV-Dynamics software.

RV shape was quantified through the calculation of mean curvature (MC) as a measure of endocardial surface bending. MC was computed for each mesh point, commonly referred to as vertex, in accordance with the methods proposed by Meyer et al. [12]. MC reflects the mean of all curvatures represented in the surface patch spanned by the vertex and its direct neighbours. Negative MC reflects a locally concave surface, zero MC a flat surface and positive MC a convex surface, as assessed from a viewpoint outside the RV [13]. To compensate for the scale-dependent nature of curvature measures, MC was normalized for RV size by dividing it by the curvature of a sphere with a volume equal to that of the RV and was presented as a percentage [14]. MC was calculated in end-diastole (ED) and end-systole (ES), defined as the frame with the largest and smallest RV volume, respectively.

RV function was quantified by means of area strain (AS) and longitudinal strain (LS). AS was computed as the percentual area change of each mesh or mesh segment between ED and ES. For the calculation of LS, a longitudinal axis was defined between the apex and midpoint between the tricuspid- and pulmonary valve, onto which endocardial shortening was projected. LS was subsequently derived by calculating the percentual change of length between ED and ES, in the direction of the longitudinal axis. While AS represents the shrinking of area patches, LS can be seen as the component of that shrinkage along the longitudinal direction.

**Fig. 1:**
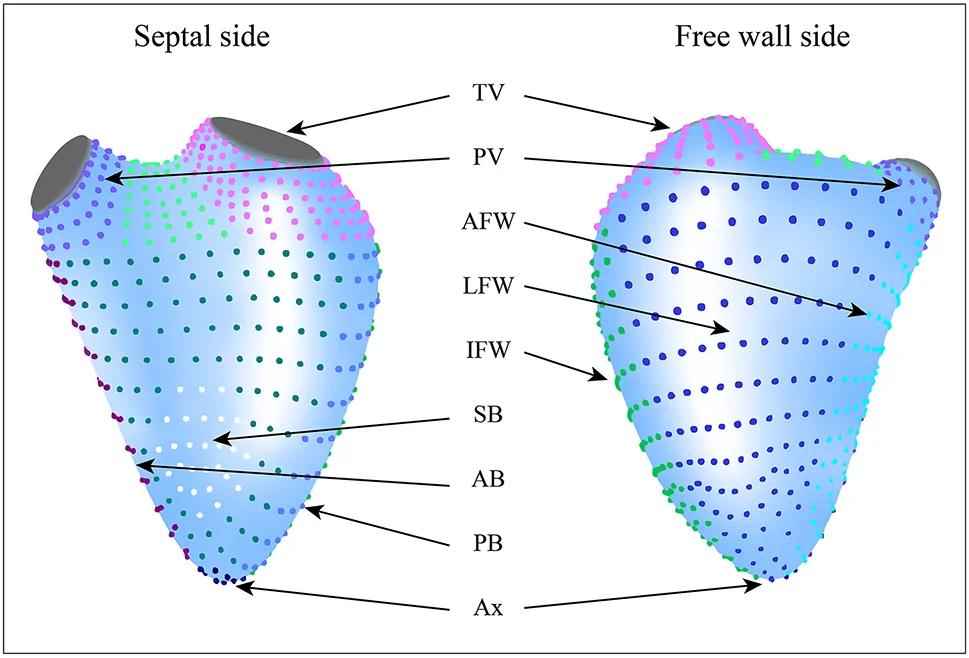
Regional division right ventricle. The 3D endocardial surface of the right ventricle was divided into the following regions: tricuspid valve (TV), pulmonary valve (PV), anterior free wall (AFW), lateral free wall (LFW), inferior free wall (IFW), septal body (SB), anterior boundary (AB), posterior boundary (PB) and apex (Ax).

All parameters were measured globally and over different segments of the RV as specified in Fig. 1. The global free wall (GFW) segment was defined, consisting of the inferior free wall (IFW), the lateral free wall (LFW) and the anterior free wall (AFW). The global septum (GSP) segment was defined with the septal body (SB), describing the central portion of the interventricular septum. The region reflecting transition from RV free wall to septal side was indicated as the global boundary (GB), including the anterior boundary (AB), posterior boundary (PB) and apex (Ax). Lastly, the tricuspid valve (TV) and pulmonary valve (PV) regions were identified, for which function analysis was not feasible due to anatomical and imaging limitations.

### Statistical analysis

ToF patients were categorized according to the presence of volume overload, pressure overload, or no overload, based on conventional echocardiographic evaluation. Comparisons were performed both between patient subgroups and with healthy controls.

Distribution of the data was examined through histograms and the Shapiro-Wilk test. When normally distributed, continuous variables were expressed as mean ± standard deviation (SD) and compared using a Student’s *t* test. Non-parametric variables were presented as median and interquartile range (IQR) and compared using a Mann-Whitney U test. Categorical variables were presented as frequencies with percentages and compared using a chi-square test. For comparison of normally distributed data between more than 2 groups, a one-way ANOVA test was applied. In the case of non-parametric data, the Kruskal-Wallis test was applied. Post-hoc analysis was performed using Bonferroni correction for multiple comparisons. RV shape and function parameters were all considered as non-parametric variables to allow for interregional comparison. Pearson’s and Spearman’s correlation coefficient were calculated to measure correlation, according to distribution. A p-value below 0.05 was considered statistically significant. Statistical analysis was performed in SPSS for Windows (Version 28.0, SPSS Inc., Chicago, IL, USA).

## Results

### Baseline characteristics

A total of 50 patients with ToF were included (median age 38 years, 36% female). An age- matched cohort of 50 healthy individuals served as a control group. Baseline patient characteristics and conventional echocardiographic parameters are summarized in Tables 1 and 2. Among ToF patients, 50% had undergone initial surgical correction with a transannular patch and 54% had undergone PVR later in life. Based on conventional echocardiographic assessment, volume overload was present in 20 patients, pressure overload in three patients and no volume or pressure overload in 27 patients. Data on the three patients with pressure overload is provided separately in Supplementary Table 1.

**Table 1:** Baseline characteristics of patients with ToF and controls

	ToF				
	All (*n* = 50)	Volume overload (*n* = 20)	Absent overload (*n* = 27)	Controls (*n* = 50)	*P*-value
Age, years	38 [22–46]	25^ab^ [21–33]	42 [35–49]	34 [25–47]	0.002
Sex, female (%)	18 (36)	6 (30)	11 (41)	16 (32)	0.678
BMI, kg/m^2^	24 [22–25]	23 [22–25]	24 [22–26]	23 [22–25]	0.325
BSA, m^2^	1.9 ± 0.2	1.9 ± 0.2	1.9 ± 0.2	1.9 ± 0.2	0.551
ECG					
QRS > 120ms, n (%)	33 (70)	14 (74)	18 (72)		0.901
RBBB, n (%)	40 (82)	17 (90)	20 (74)		0.395
RBBB with LAHB, n (%)	9 (19)	2 (11)	6 (24)		0.251
Surgical interventions					
Trans-annular patch, n (%)	25 (50)	8 (40)	15 (56)		0.292
Pulmonary valve replacement^1^, n (%)	27 (54)	2 (10)	22 (81)		< 0.001

BMI, body mass index; BSA, body surface area; ECG, electrocardiogram; RBBB, right bundle branch block; LAHB, left anterior hemiblock; ^1^Pulmonary homograft, pulmonary allograft, Melody valv*e*. ^a^*P *< 0.05 vs. control subjects. ^b^*P *< 0.05 between volume overload and absent overload.

**Table 2:** Baseline echocardiography of patients with ToF and controls

	ToF			
	Volume overload (*n* = 20)	Absent overload (*n* = 27)	Controls (*n* = 50)	*P*-value
*Two-dimensional echocardiography*				
LVEF, (%)	55^a^ [51–57]	53^a^ [49–60]	60 [57–65]	< 0.001
RV basal dimension, mm	50^a^ [45–53]	45^a^ [40–50]	40 [35–42]	< 0.001
RV longitudinal dimension, mm	95^a^ ± 9	91^a^ ± 11	85 ± 8	< 0.001
RVOT dimension, mm	39^a^ [33–49]	37^a^ [35–42]	33 [29–35]	< 0.001
RA area, cm^2^	24^a^ [19–26]	22^a^ [18–28]	17 [14–20]	< 0.001
RV S’, cm/s	12^b^ ± 2	10^ab^ ± 2	12 ± 2	< 0.001
TAPSE, mm	19^ab^ ± 5	16^ab^ ± 3	26 ± 4	< 0.001
FAC, %	34^a^ ± 7	35^a^ ± 8	43 ± 6	< 0.001
*Three-dimensional echocardiography*				
RVEF, %	49^a^ [43–54]	43^a^ [39–48]	58 [55–61]	< 0.001
RVEDV, mL	230^ab^ [164–304]	159^ab^ [117–217]	121 [97–143]	< 0.001
RVEDVi, mL/m^2^	117^ab^ [96–141]	80^ab^ [71–100]	61 [50–70]	< 0.001
RVESV, mL	109^a^ [87–160]	83^a^ [71–140]	48 [39–63]	< 0.001
RVESVi, mL/m^2^	62^a^ [48–78]	43^a^ [38–62]	24 [20–32]	< 0.001

LVEF, left ventricular ejection fraction; RV, right ventricle; RVOT, right ventricular outflow tract; RA, right atrium; TAPSE, tricuspid annular plane systolic excursion; FAC, fractional area change; RVEF, right ventricular ejection fraction; RVEDV, right ventricular end-diastolic volume; RVEDVi, right ventricular end-diastolic volume indexed for body surface area; RVESV, right ventricular end-systolic volume; RVESVi, right ventricular end-systolic volume indexed for body surface area. ^a^*P *< 0.05 vs. control subjects. ^b^*P *< 0.05 between volume overload and absent overload.

RV dimensions were significantly larger in ToF patients compared with controls, with the highest indexed RV end-diastolic volumes (RVEDVi) observed in patients with volume overload (117 mL/m^2^ [96–141] vs. 80 mL/m^2^ [71–100] vs. 61 mL/m^2^ [50–70]; for volume overload, absent overload and controls, respectively; *p* < 0.001). Conventional metrics of RV function were within normal ranges in the majority of ToF patients, however, reduced systolic function was observed in a subset of patients. This was reflected by differences in RV ejection fraction (RVEF) across both patient groups (49% [43–54] vs. 43% [39–48] vs. 58% [55–61]; for volume overload, absent overload and controls, respectively; *p* < 0.001).

### Right ventricular shape

MC values of the RV at ED, as presented in Table 3, were significantly lower in ToF patients compared with healthy controls (106% [104–109] vs. 105% [100–108] vs. 112% [108–116]; for volume overload, absent overload and controls, respectively; *p* < 0.001). Similar differences were observed at end-systole (ES). Collectively, these findings indicate a more spherical and less angular RV configuration in ToF patients.

**Table 3:** Three-dimensional shape of the right ventricle

	ToF							
	Volume overload (*n* = 20)		Absent overload (*n* = 27)		Controls (*n* = 50)		*P*-value	
	ED	ES	ED	ES	ED	ES	ED	ES
Global right ventricle	106^a^ [104–109]	107^a^ [102–111]	105^a^ [100–108]	102^a^ [97–108]	112 [108–116]	116 [112–125]	< 0.001	< 0.001
Global free wall, %	82 [80–86]	77 [73–82]	81 [79–84]	74 [72–79]	84 [78–90]	80 [74–93]	0.362	0.065
Inferior free wall, %	110 [102–115]	106^a^ [96–117]	107 [99–116]	101 [90–110]	102 [96–112]	95 [84–103]	0.095	0.038
Lateral free wall, %	80 [72–87]	75 [66–89]	78 [71–85]	76^a^ [64–86]	81 [74–87]	86 [74–104]	0.709	0.013
Anterior free wall, %	59 [50–76]	51 [40–59]	55 [51–68]	47 [41–59]	70 [55–83]	53 [43–73]	0.071	0.554
Global septum, %	5 [-2-11]	2 [-4-11]	0 [-6-4]	-1 [-10-8]	3 [-7-15]	6 [-7-21]	0.199	0.290
Septal body, %	-50^a^ [-58;-40]	-45^a^ [-53;-37]	-49 [-68;-42]	-53 [-63;-39]	-64 [-83;-52]	-62 [-77;-52]	0.007	< 0.001
Global border, %	407 [370–427]	411^a^ [391–444]	429 [398–459]	447 [389–476]	425 [389–467]	463 [411–519]	0.121	0.013
Apex, %	464^a^ [419–529]	412^a^ [377–464]	501^a^ [467–541]	459^a^ [391–490]	554 [486–619]	547 [467–669]	< 0.001	< 0.001
Anterior boundary, %	532 [470–582]	561 [496–608]	540 [484–606]	553 [480–636]	492 [441–578]	497 [439–587]	0.167	0.169
Posterior boundary, %	253^ab^ [227–265]	258^ab^ [237–291]	283^b^ [260–301]	327^ab^ [303–336]	294 [264–329]	371 [322–426]	< 0.001	< 0.001
Tricuspid valve region, %	105 [96–110]	103 [100–116]	96^a^ [86–106]	97^a^ [81–104]	107 [98–122]	115 [94–130]	0.004	0.002
Pulmonary valve region, %	118 [109–128]	114^a^ [106–123]	102^a^ [87–126]	99^a^ [79–122]	131 [115–141]	129 [114–140]	< 0.001	< 0.001

*P*-values were reported based on a Kruskal-Wallis test. Post-hoc analyses with Bonferroni correction for multiple comparisons were conducted to assess pairwise differences (^a^*P* < 0.05 vs. controls, ^b^*P* < 0.05 between volume overload and absent overload).

**Fig. 2:**
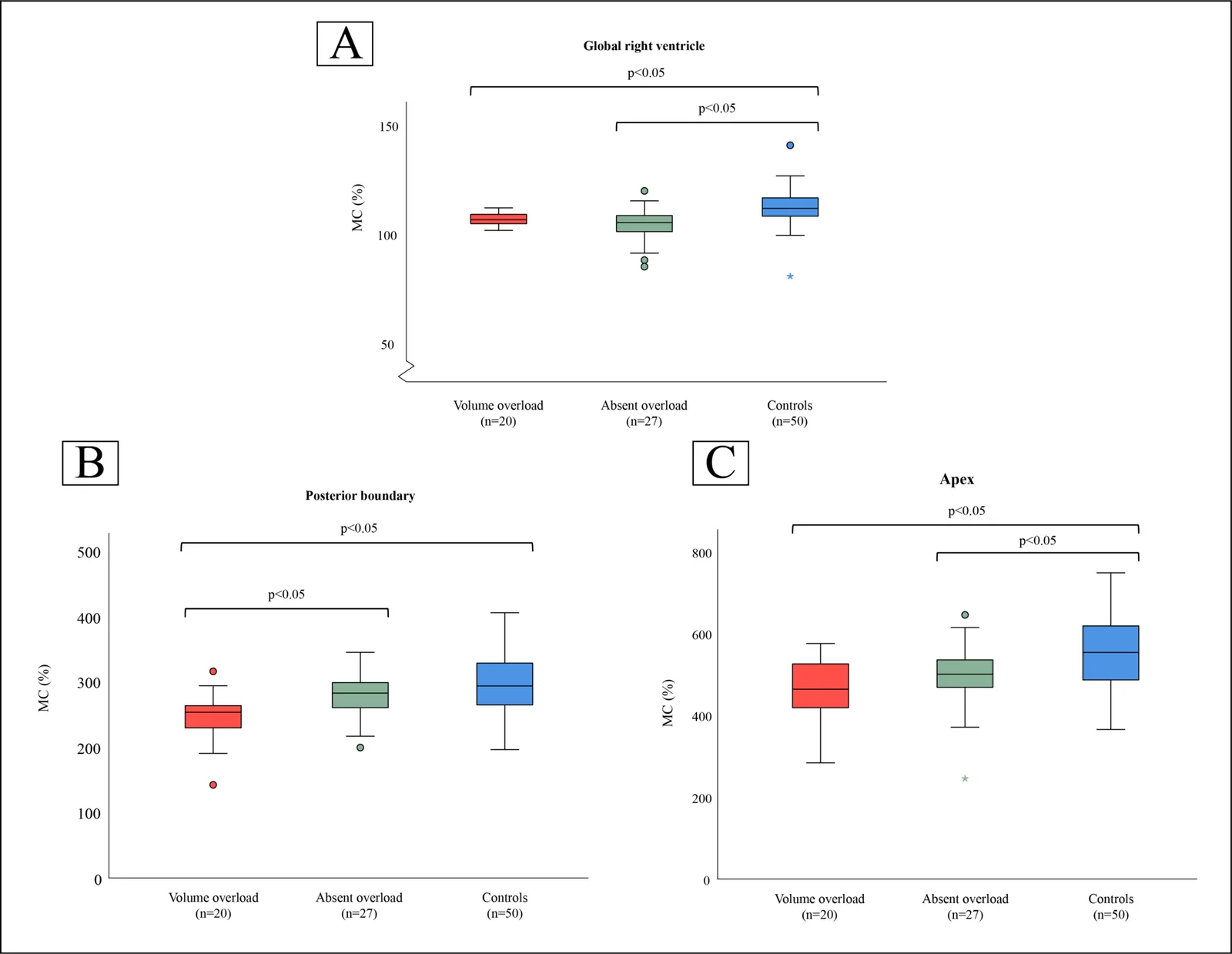
RV shape analysis. Right ventricular shape was quantified by calculating mean curvature (MC) in patients with volume overload (*red*), absent overload (*green*) and healthy controls (*blue*). Shape was quantified for the global right ventricle (**A**). Segmental analysis revealed the posterior boundary (**B**) and apex (**C**) as key regions driving right ventricular remodeling.

Segmental analysis demonstrated that the decrease in global MC was primarily driven by reductions in convexity of the PB and Ax regions. (Fig. 2) The most pronounced reductions were found in patients with volume overload, with PB MC values of 253% [227–265], compared with 283% [260–301] in patients without volume or pressure overload, and 294% [264–329] in healthy controls (*p* < 0.001). During ES, additional reductions in convexity were observed in the LFW, whereas increased convexity was observed in the IFW.

The SB region was the only RV region exhibiting concavity throughout the cardiac cycle. Although only statistically significant in patients with volume overload, both ToF subgroups demonstrated reduced concavity, consistent with septal flattening (-50% [-58;-40] vs. -49% [-68;-42] vs. -64% [-83; -52]; for volume overload, absent overload and controls, respectively; *p* = 0.007).

**Fig. 3:**
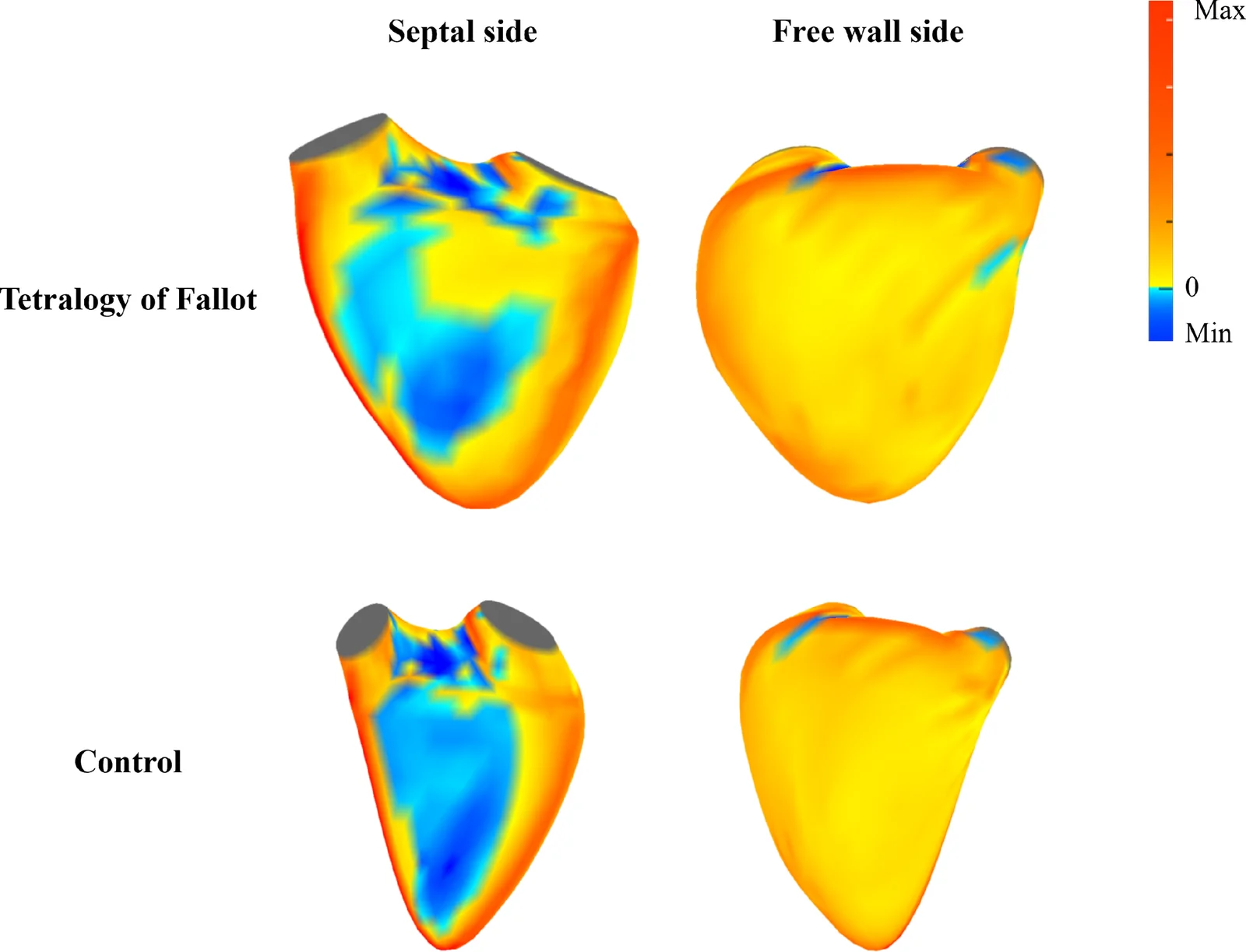
Illustrative cases RV shape analysis. Visual representation of RV shape is shown at end-diastole for an illustrative ToF patient and healthy control, displaying the septal and free wall side. The RV endocardial surface is color-coded according to MC values. In the ToF patient, decreased convexity is evident in the PB and Ax regions. Additionally, the septum shows a notable reduction in concavity, indicative of septal flattening.

A representative comparison between a ToF patient and a healthy control is shown in Fig. 3, providing a qualitative illustration of the observed differences in RV morphology.

### Right ventricular function

**Fig. 4:**
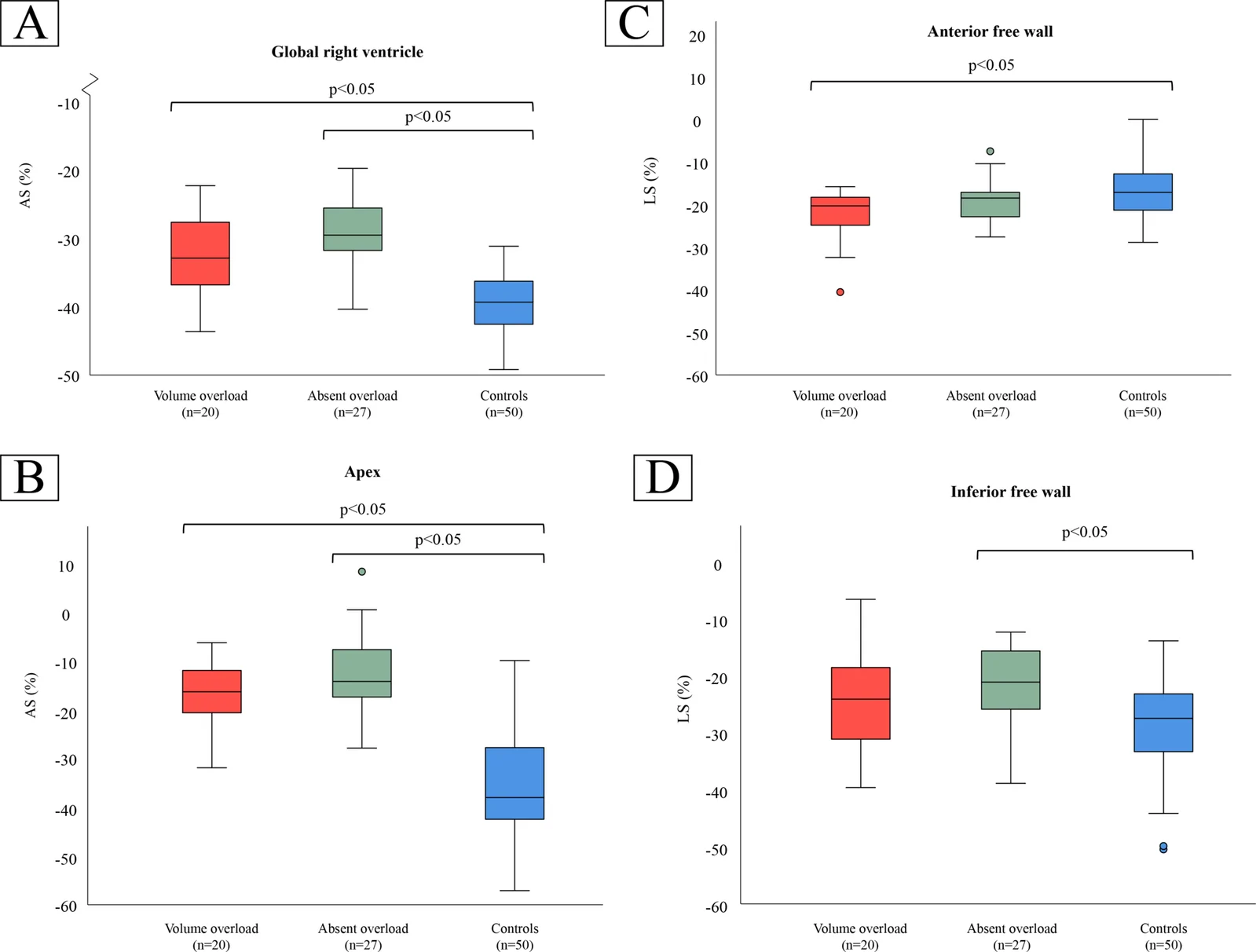
RV function analysis. RV function was analysed by measuring area strain (AS) and longitudinal strain (LS). AS for the global RV and apex are shown in **A** and **B**, respectively. Segmental analysis of longitudinal function for the anterior free wall and inferior free wall are shown in **C** and **D**, respectively. RV function analysis was performed in patients with volume overload (*red*), absent overload (*green*) and controls (*blue*).

AS, representing multidirectional myocardial deformation, was significantly reduced for the global RV in ToF patients compared with controls (*p* < 0.001; Table 4). Segmental analysis demonstrated significantly lower AS values across all RV regions, with the most pronounced reduction observed at the apex (-16% [-21;-12] vs. -14% [-17;-6] vs. -38% [-42;-28]; for volume overload, absent overload and controls, respectively; *p* < 0.001). (Fig. 4) Although not statistically significant, AS tended to be more reduced in patients without volume or pressure overload, compared with those with volume overload.

**Table 4:** Three-dimensional function of the right ventricle

	ToF			
	Volume overload (*n* = 20)	Absent overload (*n* = 27)	Control (*n* = 50)	*P*-value
*Area strain*				
Global right ventricle, %	-33^a^ [-37;-27]	-29^a^ [-32;-25]	-39 [-43;-36]	< 0.001
Global free wall, %	-37^a^ [-43;-32]	-33^a^ [-35;-30]	-44 [-46;-42]	< 0.001
Inferior free wall, %	-45 [-47;-36]	-38^a^ [-41;-31]	-46 [-49;-42]	< 0.001
Lateral free wall, %	-37^a^ [-45;-33]	-35^a^ [-38;-31]	-48 [-51;-44]	< 0.001
Anterior free wall, %	-29^a^ [-35;-24]	-25^a^ [-30;-19]	-35 [-39;-32]	< 0.001
Global septum, %	-31^a^ [-35;-24]	-30^a^ [-34;-21]	-38 [-42;-32]	< 0.001
Septal body, %	-29^a^ [-35;-23]	-29^a^ [-34;-20]	-38 [-44;-34]	< 0.001
Global border, %	-35^a^ [-39;-30]	-32^a^ [-36;-25]	-41 [-45;-36]	< 0.001
Apex, %	-16^a^ [-21;-12]	-14^a^ [-17;-6]	-38 [-42;-28]	< 0.001
Anterior boundary, %	-33^b^ [-38;-29]	-28^ab^ [-30;-25]	-33 [-39;-27]	0.002
Posterior boundary, %	-38^a^ [-43;-31]	-37^a^ [-43;-32]	-47 [-52;-42]	< 0.001
*Longitudinal strain*				
Global right ventricle, %	-17 [-21;-14]	-16^a^ [-17;-14]	-20 [-22;-16]	0.002
Global free wall, %	-21 [-25;-18]	-18^a^ [-20;-16]	-21 [-26;-16]	0.027
Inferior free wall, %	-24 [-31;-18]	-21^a^ [-27;-14]	-27 [-33;-23]	0.002
Lateral free wall, %	-17 [-23;-14]	-16^a^ [-18;-12]	-19 [-23;-15]	0.013
Anterior free wall, %	-20^a^ [-25;-18]	-18 [-23;-17]	-17 [-21;-13]	0.003
Global septum, %	-19 [-23;-16]	-19 [-21;-15]	-20 [-24;-17]	0.315
Septal body, %	-21 [-27;-17]	-21 [-25;-17]	-21 [-27;-19]	0.705
Global border, %	-22 [-27;-18]	-21 [-25;-17]	-20 [-27;-18]	0.609
Apex, %	-17 [-19;-13]	-13 [-19;-9]	-15 [-21;-9]	0.332
Anterior boundary, %	-22^a^ [-25;-18]	-20 [-24;-17]	-18 [-23;-14]	0.032
Posterior boundary, %	-23 [-28;-19]	-22 [-27;-18]	-25 [-30;-19]	0.404

*P*-values were reported based on a Kruskal-Wallis test. Post-hoc analyses with Bonferroni correction for multiple comparisons were conducted to assess pairwise differences (^a^*P *< 0.05 vs. controls, ^b^*P *< 0.05 between volume overload and absent overload).

Global LS of the RV did not differ significantly between patients with volume overload and controls; however, patients without volume or pressure overload exhibited significantly reduced LS compared with controls (-17% [-21;-14] vs. -16% [-17;-14] vs. -20% [-22;-16]; for volume overload, absent overload and controls, respectively; *p* = 0.002). Segmental analysis indicated that this reduction was mainly driven by reduced LS in the IFW and LFW regions. In contrast, patients with volume overload demonstrated increase of LS in the AFW and AB regions (*p* < 0.05).

An overview of shape and function parameters comparing patients with a native pulmonary valve and post-PVR is provided in Supplemental Tables 2 and 3.

**Fig. 5:**
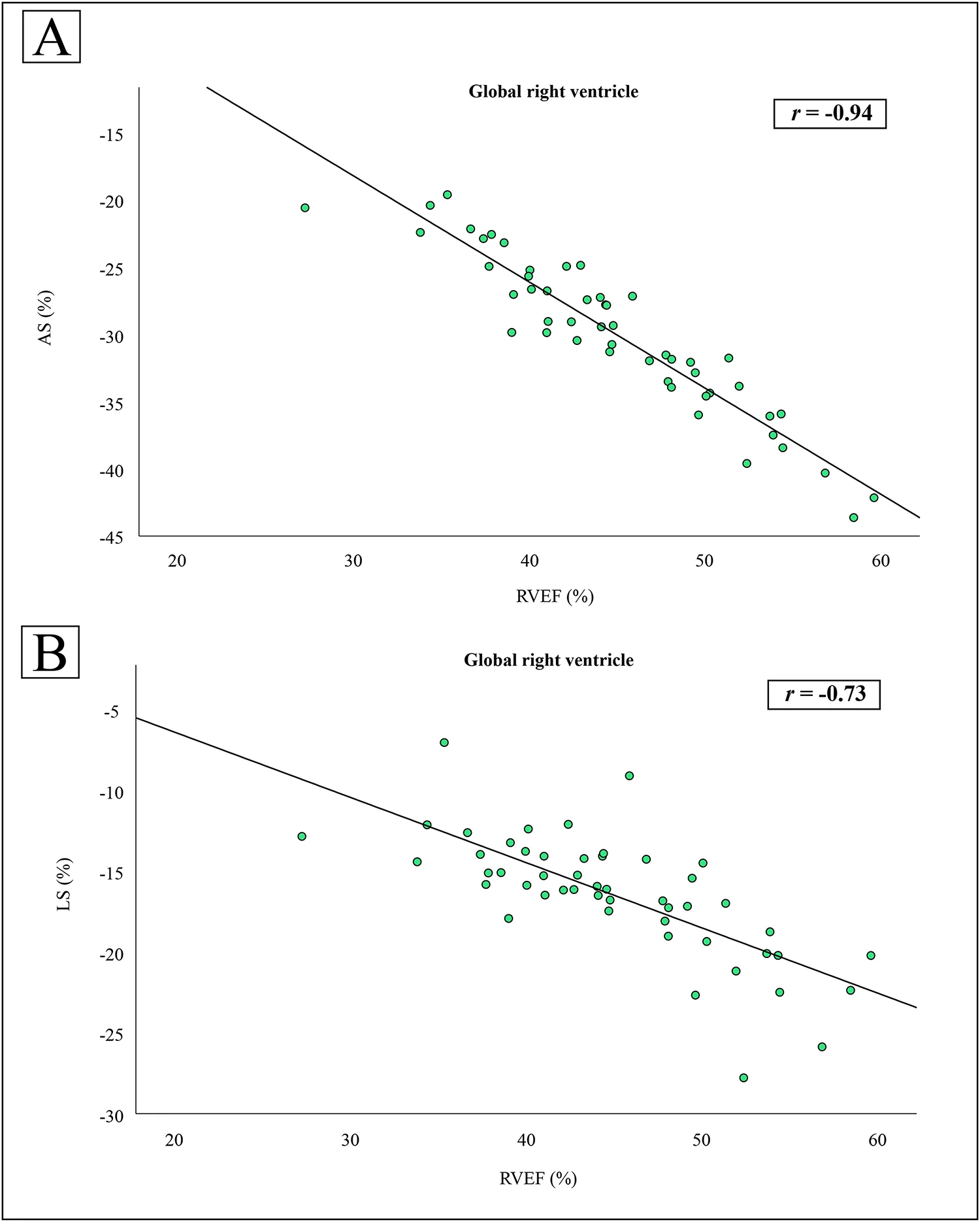
Correlation conventional echocardiography. The relation between area strain (AS) and longitudinal strain (LS) for the global RV with right ventricular ejection fraction (RVEF) is visualized in scatterplots **A** and **B**, respectively.

A moderate to strong inverse correlation was observed between AS and RVEF, with correlation coefficient *r* of -0.94 for global RV AS (*p* < 0.05). (Fig. 5) Correlations with other conventional echocardiographic parameters were generally weaker, despite moderate correlations with AS of the RV free wall.

Longitudinal strain showed moderate correlations with RVEF (*r *= -0.73 for global RV LS; *p* < 0.05), but weaker associations with other traditional RV function measures. (Supplemental Table 4)

## Discussion

The aim of this study was to explore 3D RV shape and function in patients with ToF using a novel, in-house developed software tool applied to standard 3DE imaging. Our results demonstrate that curvature-based analysis provides a region-specific characterization of RV remodeling, distinguishing a more spherical and less convex (angled) RV geometry in ToF patients compared to healthy controls. The posterior boundary and apex emerged as key regions involved in RV remodeling, highlighting their potential value as markers for disease progression in patients with chronic RV loading. Furthermore, comprehensive assessment of global and regional RV function using 3DE-derived AS and LS offered insights into the mechanisms underlying RV dysfunction in ToF.

### Right ventricular shape

Cardiac remodeling encompasses changes in ventricular shape, size and function. While current diagnostic approaches allow increasingly refined assessment of RV size and function, objective quantification of RV shape remains challenging due to its complex geometry. By leveraging the regional and 3D nature of 3DE, RV geometry can be quantified using mean curvature, providing a direct measure of local convexity and concavity. From a biomechanical perspective, mean curvature may reflect underlying wall stress distribution, as RV wall stress is proportional to intracavitary pressure and local radius and inversely related to wall thickness, consistent with the thin-wall theory.

In our ToF population, global RV curvature was significantly reduced, in line with RV dilatation and chronic volume overload. Regional curvature analysis revealed that these changes were predominantly driven by reduced convexity of the PB and Ax regions, identifying these areas as principal contributors to RV remodeling.

The PB showed pronounced flattening in patients with volume overload, likely reflecting increased wall stress and outward deformation secondary to chronic pulmonary regurgitant. Although regional RV curvature has not been extensively studied previously, RV shape visualization in ToF by Bidviene et al. [15], suggests comparable shape alterations of a segmentation analogous to our PB region. Together these findings underscore the potential of 3DE for detailed shape analysis, offering potential valuable markers for disease monitoring in the volume overloaded RV.

Apical flattening was another prominent morphological feature in our cohort and is consistent with observations from both imaging and post-mortem studies in ToF [16]. This remodeling pattern may be related to the presence of an additional circumferential myocardial fiber layer described in ToF, as described by Sanchez-Quintana et al. [17], which may constrain deformation of the mid free wall while promoting dilatation of apical and basal regions. This fiber may underlie the distinct RV remodeling pattern in ToF, where predominant RV shape alterations are confined to the apical and basal regions.

In contrast to the convex regions of the RV, the septal body remained concave throughout the entire cardiac cycle in both patients and controls. However, ToF patients displayed significantly reduced septal concavity, consistent with septal flattening, a hallmark of chronic RV volume-overload. While minimal convexity or a flat septum have also been reported in other studies, these discrepancies are likely due to differences in methodology, emphasizing the need for standardized approaches to improve reproducibility and ensure comparability across studies [15, 16, 18–20]. 

Although cautious interpretation of basal regions is warranted due to imaging limitations, reduced convexity was observed in the TV and PV regions. Basal rounding at the TV may reflect bulging associated with tricuspid regurgitation and annular dilatation in ToF [16]. Notably, this finding was observed exclusively in the absent overload group, where the majority had previously undergone PVR. Lastly, reduced convexity at the PV region likely reflects geometric consequences of transannular patch implantation during initial repair.

### Right ventricular function

Cardiac magnetic resonance (CMR) is currently considered to be the ‘gold standard’ for assessing RV function, but its availability and cost limit widespread use. 3DE offers a more accessible alternative, enabling full-volume functional assessment without reliance on geometric assumptions [21]. 

AS, which incorporates deformation in longitudinal, circumferential, and radial direction, was globally and regionally reduced in ToF patients and showed a very strong inverse correlation with RV ejection fraction (*r* = − 0.94). This strong association underscores AS as a robust marker of global RV systolic performance. Notably, the apex exhibited the greatest impairment in AS, mirroring the pronounced morphological alterations observed in this region. As conventional RV metrics predominantly reflect basal and free wall motion, these apical abnormalities likely remain undetected by standard measures, highlighting the added value of 3DE-derived AS in capturing complex RV mechanics, that may even hold prognostic value [22]. 

Longitudinal strain analysis further revealed distinct functional patterns across loading conditions. In patients with volume overload, global LS was preserved, with evidence of compensatory increases in longitudinal deformation of anterior free wall regions. In contrast, patients without volume or pressure overload demonstrated reduced global and regional LS, suggesting loss of compensatory reserve and progression toward irreversible dysfunction. These subtle shifts in contraction mechanics were not apparent on conventional echocardiography, emphasizing the enhanced sensitivity of 3DE-derived LS.

### Timing of pulmonary valve replacement

The optimal timing of PVR in ToF remains challenging, balancing the risks of early intervention (e.g. endocarditis, reintervention) against those of late intervention (irreversible RV damage, arrhythmias). In our study, curvature changes of the PB and Ax appeared to reflect the immediate impact of volume overload, with greatest loss of convexity observed in patients with present volume overload, while those without overload showed a trend toward the more curved RV configuration seen in healthy controls. While this trend in shape alterations was not clearly matched by a decline of conventional function metrics, future research is needed to prospectively evaluate RV shape analysis as marker for early-detection and its potential to improve risk stratification.

Despite the absence of statistically significant differences between subgroups, both AS and LS tended to be more impaired in patients without overload. The combination of reduced AS and loss of longitudinal function in this group supports the concept that irreversible functional decline may follow a phase of geometric adaptation and mechanical compensation. These findings reinforce the potential role of 3DE-based RV shape and function analysis in improving risk stratification and informing timing of intervention.

### Limitations

This study is limited by the limitations inherent to echocardiography of the RV. Due to the retrosternal position in the thorax, the anterior portion of the free wall is regularly overshadowed by lung or bone tissue, leading to poor imaging quality of these regions. Shape and function analyses of these regions, therefore rely on less precise information. Additionally, only patients with moderate to good quality 3DE studies were considered for inclusion in this study. Analyses performed in this study may, therefore, not be applicable to all ToF patients.

Moreover, analysis of RV shape and function using our custom software, relies on accurately generated 3D endocardial surface models. These models were acquired using a semi-automatic segmentation software application, requiring manual interactions and fine tuning. Despite the high level of expertise in our center with this technique, the results were still sensitive to operator dependent variations and inaccuracies involving complex segmentations such as the TV and PV region.

Lastly, a relatively healthy cohort of ToF patients was studied. Future research should focus on more advanced stages of disease to investigate the prognostic value of these newly derived 3DE metrics.

## Conclusions

A novel software tool was developed for the quantification of global and regional RV shape and function using standard 3DE datasets. Curvature analysis offers a promising method for characterizing RV remodeling, while AS and LS provide detailed insights into 3D contraction mechanics. Together, this approach allows for a comprehensive, geometry-based assessment of the RV, facilitating detection of subtle dysfunction not captured by conventional parameters. Although applied here in patients with ToF, the software is broadly applicable and may advance RV assessment across a wide range of cardiac diseases.

## Supplementary Information

Below is the link to the electronic supplementary material.

Supplementary Material

## Data Availability

Data are available upon reasonable request.
