# Supplementary material for: Exploring three-dimensional right ventricular shape and function in Tetralogy of Fallot patients
Source: Int J Cardiovasc Imaging. 2026 Jul 29;42(11):2147–58. doi: 10.1007/s10554-026-03781-y (PMC13745422; doi:10.1007/s10554-026-03781-y)
Supplement: Supplementary file 1 [file 10554_2026_3781_MOESM1_ESM.docx]

**SUPPLEMENTAL MATERIAL**

| **Supplemental Table 1.** Shape and function for three ToF patients with pressure overload | | | | |
| --- | --- | --- | --- | --- |
|  | MC-ED | MC-ES | AS | LS |
| Global right ventricle, % | 96 | 94 | -23 | -15 |
| Global free wall, % | 81 | 80 | -27 | -19 |
| Inferior free wall, % | 113 | 108 | -33 | -22 |
| Lateral free wall, % | 82 | 84 | -30 | -13 |
| Anterior free wall, % | 51 | 42 | -17 | -17 |
| Global septum, % | 4 | 3 | -24 | -17 |
| Septal body, % | -54 | -47 | -24 | -20 |
| Global border, % | 407 | 412 | -20 | -18 |
| Apex, % | 456 | 444 | -13 | -12 |
| Anterior boundary, % | 563 | 521 | -20 | -19 |
| Posterior boundary, % | 259 | 290 | -25 | -22 |
| Tricuspid valve region, % | 73 | 62 |  |  |
| Pulmonary valve region, % | 95 | 80 |  |  |
| MC-ED, mean curvature at end-diastole; MC-ES, mean curvature at end-systole; AS, area strain; LS, longitudinal strain. | | | | |

| **Supplemental Table 2.** Comparison 3D RV shape between patients with native pulmonary valve and post-PVR | | | | | | | |
| --- | --- | --- | --- | --- | --- | --- | --- |
|  | Native valve  (n=23) | |  | Post-PVR  (n=27) | | *P*-value | |
|  | ED | ES |  | ED | ES | ED | ES |
| Global right ventricle, % | 106 [103-109] | 106 [102-110] |  | 105 [100-108] | 102 [97-108] | 0.397 | 0.307 |
| Global free wall, % | 81 [80-85] | 76 [72-80] |  | 81 [80-86] | 76 [72-83] | 0.977 | 0.748 |
| Inferior free wall, % | 107 [101-113] | 108 [93-116] |  | 112 [99-117] | 101 [92-110] | 0.620 | 0.386 |
| Lateral free wall, % | 80 [74-88] | 75 [68-88] |  | 78 [70-81] | 76 [64-86] | 0.189 | 0.823 |
| Anterior free wall, % | 55 [48-64] | 45 [35-57] |  | 56 [52-72] | 48 [42-71] | 0.365 | 0.153 |
| Global septum, % | 4 [-4-11] | 1 [-5-11] |  | 1 [-6-5] | 2 [-10-11] | 0.170 | 0.676 |
| Septal body, % | -49 [-60;-41] | -46 [-56;-37] |  | -52 [-68;-42] | -50 [-63;-39] | 0.808 | 0.239 |
| Global border, % | 411 [377-451] | 434 [400-450] |  | 412 [398-454] | 442 [387-460] | 0.633 | 0.620 |
| Apex, % | 471 [420-531] | 413 [383-474] |  | 493 [463-526] | 446 [387-483] | 0.477 | 0.527 |
| Anterior boundary, % | 542 [490-614] | 565 [504-620] |  | 521 [464-590] | 521 [452-572] | 0.419 | 0.108 |
| Posterior boundary, % | 255 [235-270] | 258 [244-306] |  | 276 [259-294] | 321 [290-346] | 0.023 | 0.004 |
| Tricuspid valve region, % | 101 [91-110] | 102 [96-115] |  | 97 [88-108] | 97 [81-104] | 0.355 | 0.096 |
| Pulmonary valve region, % | 115 [105-124] | 113 [103-122] |  | 102 [89-126] | 104 [81-122] | 0.231 | 0.280 |
| PVR, pulmonary valve replacement; ED, end-diastole; ES, end-systole. | | | | | | | |

| **Supplemental Table 3.** Comparison 3D RV function between patients with native pulmonary valve and post-PVR | | | |
| --- | --- | --- | --- |
|  | Native valve  (n=23) | Post-PVR  (n=27) | *P*-value |
| Area strain | | | |
| Global right ventricle, % | -32 [-36;-28] | -27 [-31;-25] | 0.025 |
| Global free wall, % | -37 [-43;-33] | -32 [-35;-29] | 0.010 |
| Inferior free wall, % | -44 [-47;-36] | -36 [-41;-31] | 0.019 |
| Lateral free wall, % | -38 [-44;-34] | -34 [-38;-30] | 0.060 |
| Anterior free wall, % | -28 [-32;-24] | -23 [-30;-17] | 0.055 |
| Global septum, % | -31 [-35;-25] | -27 [-33;-21] | 0.131 |
| Septal body, % | -29 [-36;-25] | -28 [-34;-21] | 0.189 |
| Global border, % | -35 [-38;-30] | -32 [-36;-25] | 0.096 |
| Apex, % | -14 [-20;-10] | -15 [-18;-11] | 0.778 |
| Anterior border, % | -33 [-37;-28] | -27 [-30;-20] | 0.007 |
| Posterior border, % | -39 [-43;-33] | -36 [-43;-28] | 0.386 |
| Longitudinal strain | | | |
| Global right ventricle, % | -17 [-20;-14] | -15 [-17;-14] | 0.055 |
| Global free wall, % | -20 [-24;-18] | -18 [-20;-16] | 0.026 |
| Inferior free wall, % | -24 [-31;-17] | -21 [-27;-18] | 0.271 |
| Lateral free wall, % | -17 [-23;-14] | -16 [-18;-12] | 0.100 |
| Anterior free wall, % | -21 [-24;-18] | -18 [-23;-17] | 0.040 |
| Global septum, % | -19 [-24;-17] | -17 [-21;-15] | 0.196 |
| Septal body, % | -21 [-27;-17] | -21 [-24;-17] | 0.477 |
| Global border, % | -23 [-27;-18] | -20 [-24;-17] | 0.224 |
| Apex, % | -16 [-18;-12] | -13 [-20;-9] | 0.553 |
| Anterior boundary, % | -23 [-26;-18] | -19 [-24;-17] | 0.189 |
| Posterior boundary, % | -24 [-27;-18] | -22 [-27;-18] | 0.465 |
| PVR, pulmonary valve replacement. | | | |

| **Supplemental Table 4.** Correlation between traditional echocardiography and 3D RV function in patients with ToF | | | | | | |
| --- | --- | --- | --- | --- | --- | --- |
|  | RVEF | TAPSE | FAC | S’ | LVEF | QRS |
| Area strain |  |  |  |  |  |  |
| Global right ventricle | **-0.94** | **-0.48** | **-0.40** | **-0.40** | **-0.41** | 0.08 |
| Global free wall | **-0.93** | **-0.56** | **-0.35** | **-0.47** | **-0.47** | 0.08 |
| Inferior free wall | **-0.74** | **-0.60** | **-0.35** | **-0.44** | **-0.44** | 0.15 |
| Lateral free wall | **-0.84** | **-0.40** | -0.28 | **-0.38** | **-0.47** | 0.08 |
| Anterior free wall | **-0.70** | **-0.37** | **-0.31** | **-0.35** | -0.23 | -0.00 |
| Global septum | **-0.74** | -0.26 | **-0.29** | -0.23 | -0.20 | 0.00 |
| Septal body | **-0.66** | -0.27 | -0.24 | -0.26 | -0.24 | -0.03 |
| Global border | **-0.85** | **-0.34** | **-0.42** | **-0.29** | **-0.46** | 0.24 |
| Apex | **-0.36** | -0.08 | 0.06 | -0.18 | -0.25 | 0.02 |
| Anterior boundary | **-0.76** | **-0.49** | **-0.31** | **-0.42** | **-0.48** | -0.03 |
| Posterior boundary | **-0.73** | -0.24 | **-0.47** | -0.05 | **-0.35** | **0.34** |
| Longitudinal strain |  |  |  |  |  |  |
| Global right ventricle | **-0.73** | **-0.43** | -0.17 | **-0.33** | **-0.35** | -0.13 |
| Global free wall | **-0.69** | **-0.53** | -0.11 | **-0.34** | **-0.37** | -0.11 |
| Inferior free wall | **-0.46** | **-0.51** | -0.03 | **-0.36** | **-0.33** | -0.00 |
| Lateral free wall | **-0.50** | **-0.30** | -0.09 | -0.11 | -0.28 | -0.27 |
| Anterior free wall | **-0.76** | **-0.43** | **-0.50** | **-0.31** | **-0.32** | 0.06 |
| Global septum | **-0.66** | **-0.37** | -0.28 | **-0.31** | **-0.32** | 0.02 |
| Septal body | **-0.59** | **-0.29** | **-0.29** | -0.27 | **-0.38** | 0.05 |
| Global border | **-0.56** | **-0.48** | **-0.31** | **-0.33** | **-0.33** | -0.01 |
| Apex | -0.23 | -0.22 | -0.24 | -0.24 | -0.23 | -0.19 |
| Anterior boundary | **-0.60** | **-0.45** | **-0.32** | -0.27 | **-0.38** | -0.06 |
| Posterior boundary | **-0.46** | **-0.48** | -0.27 | **-0.33** | -0.24 | 0.06 |
| RVEF, right ventricular ejection fraction; TAPSE, tricuspid annular plane systolic excursion; FAC, fractional area change; LVEF, left ventricular ejection fraction. Correlations statistically significant at 0.05 level in **bold**. | | | | | | |
